# The long exercise test as a functional marker of periodic paralysis

**DOI:** 10.1002/mus.27465

**Published:** 2021-12-06

**Authors:** Ana Ribeiro, Karen J Suetterlin, Iwona Skorupinska, S.Veronica Tan, Jasper M Morrow, Emma Matthews, Michael G Hanna, Doreen Fialho

**Affiliations:** 1 Department of Clinical Neurophysiology, King’s College Hospital, London, UK; 2 Queen Square Centre for Neuromuscular Diseases, University College London, UK; 3 Department of Clinical Neurophysiology, Royal Victoria Infirmary, Newcastle Upon Tyne, UK; 4 Department of Neurology and Neurophysiology, St Thomas’ Hospital, Guy’s and St Thomas’ NHS Foundation Trust and Department of Academic Neurosciences, Kings College London, UK; 5 Atkinson-Morley Neuromuscular Centre, St George’s University Hospitals NHS Foundation Trust, London, UK

**Keywords:** Periodic paralysis, Long exercise test (McManis), Electrodiagnostic study, Sensitivity, Decrement

## Abstract

**Introduction/Aims:**

The aim of this study was to demonstrate sensitivity of the long exercise test (LET) in the diagnosis of periodic paralysis (PP) and establish correlation with clinical phenotype and genotype.

**Methods:**

From an unselected cohort of 335 patients who had an LET we analysed 67 patients with genetic confirmation of PP and/or a positive LET.

**Results:**

32/45 patients with genetically confirmed PP had a significant decrement after exercise (sensitivity of 71%). Performing the short exercise test before the LET in the same hand confounded results in 4 patients. Sensitivity was highest in patients with frequent (daily or weekly) attacks (8/8, 100%), intermediate with up to monthly attacks (15/21, 71%) and lowest in those with rare attacks (9/16, 56%) (p=0.035, Mann-Whitney U test). Patients with a positive LET without confirmed PP mutation comprised those with typical PP phenotype and a group with atypical features.

**Discussion:**

In our cohort, the LET is strongly correlated with the frequency of paralytic attacks suggesting a role as a functional marker. A negative test in the context of frequent attacks makes a diagnosis of PP unlikely but it does not rule out the condition in less severely affected patients.

## Introduction

The primary periodic paralyses (PP) are characterised by episodic muscle weakness and include hypokalemic PP (hypoPP), hyperkalemic PP (hyperPP) and Andersen-Tawil syndrome (ATS) due to mutations in voltage gated ion channels ^1,2^. The diagnosis is based on clinical presentation supported by genetic and electrophysiological findings but can be challenging when clinical and laboratory information do not agree ^3^. Genetic testing has been the gold standard for diagnosis, but a pathogenic mutation is found in only 60% to 70% of patients meeting clinical criteria for PP ^1^. The literature suggests that the long exercise test (LET) is highly sensitive and specific ^4–10^. The aim of this study was to demonstrate sensitivity in our cohort and correlate electrophysiology with phenotype and genotype.

## Methods

A retrospective analysis of patients attending the National Muscle Channelopathy Service from 2009 to 2017 was performed to identify all patients who had undergone an LET. The study was registered and approved as part of a clinical audit with the National Hospital for Neurology and Neurosurgery Audit Committee. Because the data was obtained during routine clinical care and collected from hospital electronic records as part of a clinical audit, such evaluations do not require patient consent. Patients were selected if they fulfilled at least one criteria: a positive LET or a pathological variant in a PP associated gene. Some patients in this study were included in a previous publication which also contained data of normal controls for the LET ^7^. Patients were subdivided into 3 main groups: a) genetics and LET positive, b) genetics positive and LET negative and c) genetics negative and LET positive. Frequency of attacks was categorised as 1) none or rare (0-1/year, this included patients with progressive weakness and no attacks), 2) moderate (>1/year to monthly) and 3) frequent (weekly to daily).

### Genetics

Patients were considered to have genetically confirmed PP if pathogenic PP mutations were found in relevant genes (*CACNA1S*, *KCNJ2*, *SCN4A*, *RYR1*). Testing was done by direct Sanger sequencing (hotspot testing in earlier cohorts or family mutations) or next generation sequencing using a ‘muscle channel panel’.

### Neurophysiology

The LET was performed as described by McManis et al ^4^. CMAPs (compound muscle action potentials) from abductor digiti minimi were monitored at baseline, during five minutes of exercise and for 50 minutes post-exercise. The same electrodes were used during short exercise test (SET), which preceded the LET, when applicable with a maximum interval between SET and LET of 30min. A post-exercise decrement of more than 40% from peak CMAP amplitude indicated a positive test. As we previously observed a significant decrement occurring after SET (3x10sec bouts of exercise separated by 1 minute), we considered the LET positive if there was >40% decrement between peak SET amplitude and the post-LET exercise CMAP. We used the largest SET CMAP amplitude to calculate the overall decrement.

### Analysis

Sensitivity was calculated using genetic testing as the gold standard. Statistical analysis was conducted using *SPSS Statistics* for Macintosh, Version 25.0. (IBM, Armonk, NY). For individual tests see table 1 and supplement 1. The level of statistical significance was set at *p*<0.05.

## Results

### Patient characteristics

**Figure 1:**
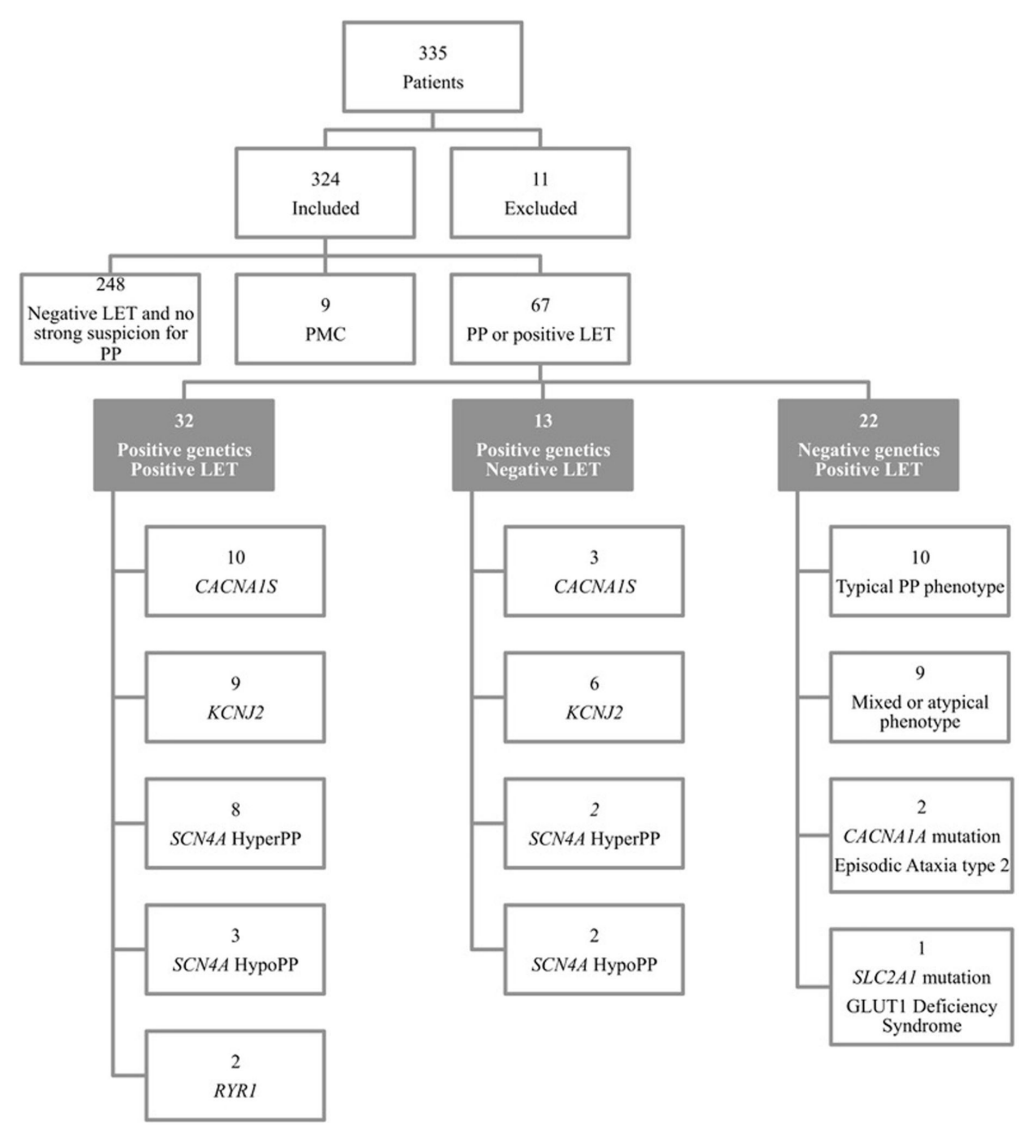
Study sample. There was no statistically significant correlation between the long exercise test (LET) results and genotype (X^2^ p=0.625). Patients with Paramyotonia congenita (PMC) were not included in the main sample as the LET pattern is often different from periodic paralysis and a positive LET is not a diagnostic criterium for this condition, which has a typical response pattern on SET particularly after cooling.

We identified 335 patients who had undergone the LET of which 248 had neither positive LET nor PP genetics. The presenting symptom was intermittent paralysis and/or progressive muscle weakness. Analysis focussed on 67 patients who had a positive LET and/or genetically confirmed PP (Figure 1, Table 1). A total of 35 patients had an SET prior to the LET. In 6 of these patients the calculated overall decrement was >5-10% larger and in 9 patients >10% larger than the decrement based on LET data alone, leading to re-classification of 4 individuals from LET negative to LET positive. LET sensitivity was 71% and did not significantly differ between genotypes, although subgroups were small. Estimated LET specificity for genetically confirmed PP was 92.4% (268/290) in the entire cohort undergoing an LET.

### Attack Frequency and LET result

**Figure 2:**
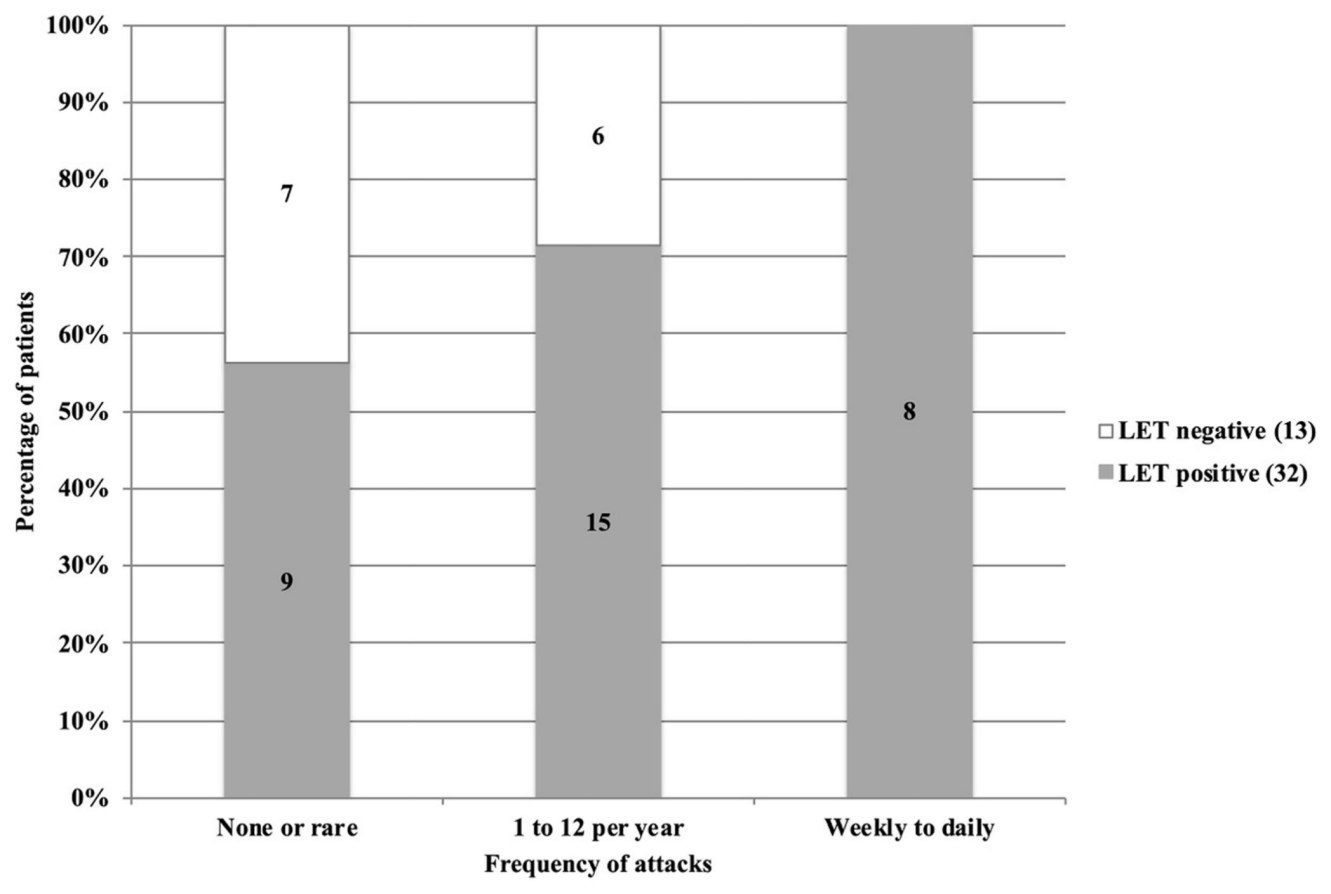
Frequency of attacks in patients with genetically confirmed periodic paralysis (PP) and correlation with long exercise test (LET) results, p=0.035 (Mann-Whitney U test)

LET results correlated with frequency of attacks (p=0.035, Mann-Whitney U test, Figure 2). More genetically confirmed PP patients with a positive LET result were on PP treatment (20/32, 62.5%) compared to those with a negative LET (3/13, 23.1%), *p* = 0.016.

### Positive LET but negative PP genetics

Typical PP features were seen in 10/22 patients with a positive LET but negative PP genetics. 9/10 patients with typical PP phenotype had negative genetic results after next generation sequencing of *CACNA1S*, *KCNJ2* and *SCN4A genes*. The nine patients with a mixed or atypical phenotype presented with exercise-induced fatigue with stiffness and cramps or exercise intolerance and fatigue.

### LET parameters and variability

16/67 patients had a repeat LET with a mean test-to-test intra-individual CMAP amplitude decrement variability of 12% (range 2%-27%). There was no clear relationship between medication changes and result of the repeat LET. Attack frequency data at the time of repeat testing was not available in sufficient numbers for analysis.

## Discussion

Our study demonstrates that the sensitivity of the LET is strongly correlated with disease activity. All patients with frequent attacks had a positive LET compared to just over half of those with rare attacks or progressive weakness. This confirms that patients who have more frequent spontaneous attacks of weakness are more likely to have an attack following a standardised provocation manoeuvre. It is also useful in clinical practice as it indicates patients with frequent attacks but negative LET are unlikely to have PP, whereas those with rare attacks and negative LET should be considered for genetic testing if clinical suspicion is strong. Our findings correlate with the observation by Tengan et al ^11^ who investigated 14 individuals from two families with hypoPP and four individuals with thyrotoxic periodic paralysis and found a positive LET only in patients with recent PP attacks while individuals with no recent attacks had a negative LET. We show that this can be applied to an unselected PP cohort of hypoPP, hyperPP and ATS. It is known that women with PP are often less severely affected and more likely to present with progressive weakness ^8–11^, which correlates with the larger number of women in the negative LET group in our study.

Even patients with rare discrete attacks of paralysis may report frequent fluctuations of strength and this could explain why a number of patients in this group still have a positive LET. Indeed a positive LET may indicate a treatable element of their condition, which can be difficult to distinguish clinically from the progressive myopathy that develops later in the disease. This hypothesis could be investigated by correlating LET with muscle MRI as a means of differentiating between reversible oedema versus irreversible fatty infiltration.

In several patients the SET confounded LET results by inducing a significant decrement on its own. Our practice is to perform the SET at room temperature and after cooling in one hand and the LET in the other hand if both are required.

The sensitivity of 71% seen in this study is within the lower range of published data (71% to 95% ^5–7^). This may reflect identification of patients with milder disease in more recent years due to better recognition of the clinical phenotype and wider availability of comprehensive genetic testing.

Previous studies have focused on improving the sensitivity of the LET including lowering the upper limits of normal ^9^. Simmons et al ^12^ applied Bayesian principles and elegantly showed that a higher sensitivity/specificity may be achieved using different cut-off values by incorporating pre-test probabilities. In practice this approach may be difficult if patients are seen by clinicians unfamiliar with these rare disorders.

In patients with typical PP phenotype but negative genetics secondary causes of PP should be considered but none were identified in our cohort. Patients with a positive LET, negative PP genetics and atypical phenotype are an interesting group with likely heterogeneous underlying pathology. Two of the patients had mutations in the *CACNA1A* gene, encoding a calcium channel expressed in the central and peripheral nervous system and linked to episodic ataxia ^13^. A patient presenting with paralytic attacks and a novel *CACNA1A* mutation but with a negative long exercise test has been reported recently ^14^. We now routinely screen patients with strong clinical evidence of periodic paralysis and negative PP genetics for *CACNA1A* mutations.

Limitations of this study included incomplete clinical data due to the retrospective nature, small subgroups, different operators and equipment over time potentially introducing variability.

We propose a re-appraisal of the role of the LET in the diagnosis of primary PP in the context of improved access to genetic evaluation. In the past, it was used as a standard diagnostic test to rule-in or rule-out the diagnosis. In patients with frequent attacks this remains a useful strategy but in those with less severe or less frequent episodes of weakness and/or atypical clinical features caution is needed in interpreting a negative LET. Our data suggests that the LET can be regarded as a functional marker of PP and in patients who describe fixed but fluctuating weakness it may indicate benefit can still be derived from pharmacological therapy.

## Supplementary Material

Supplementary 1

## Figures and Tables

**Table 1:** Results of patients with positive genetics for periodic paralysis and/or positive long exercise test.

Variables	Genetics positive LET positive	Genetics positive LET negative	Genetics negative LET positive	*p* value	Statistical test
**Patients**	**n = 32**	**n = 13**	**n = 22**	**N/A**	**N/A**
**Age in years**	36.6±14.2 (15-64)	44.4±15.4 (18-77)	36.4±12.8 (17-63)	**0.197**	**ANOVA**
**Gender M/F (M:F ratio)**	26/6 (4.3 : 1)	8/5 (1.6 : 1)	21/1 (21 : 1)	**0.040**	**Fisher**
**No of patients on PP medication (%)**	20 (62.5%)	3 (23.1%)	2 (9.1%)		
**Largest CMAP in mV**	8.1±1.8* (4.2-11.6)	9.1±1.8 (4.4-12.8)	10.5±2.1* (6.0-15.2)	**<0.001**	**ANOVA**
**Increment in %**	28.4±26.4 (-11-119)	11.8±15.1 (-13-48)	28.1±14.9 (4-58)	**0.017**	**Kruskal-Wallis**
**Decrement from peak in %**	55.0±11.6 (33-80)	28.7±6.4 (15-39)	51.3±8.4 (42-68)	**N/A†**	**N/A**
**Decrement from baseline in %**	41.8±19.1 (-12-81)	2O.1±11.2 (0-38)	37.9±12.2 (12-57)	**N/A†**	**N/A**
**Time to 40% decrement in minutes**	21.4±13.0 ‡ (4-54)	N/A	28.7±12.6 (3-46)	**0.051**	***t***

Continuous quantitative data were plotted as mean ± standard deviation (range).

* Groups with statistically significant differences

† Statistical analysis was not performed as group allocation was according to peak decrement.

‡ 4/32 patients incorporated after SET calculations could not be included as a 40% decrement was not achieved during LET. PP: periodic paralysis; CMAP: compound muscle action potential; F: female; LET: long exercise test; M: male; N/A: not applicable

## Abbreviations

ATS: Andersen-Tawil syndrome
CMAP: compound muscle action potential
F: female
HyperPP: hyperkalaemic periodic paralysis
HypoPP: hypokalaemic periodic paralysis
LET: long exercise test
M: male
N/A: not applicable
PP: periodic paralysis
SD: standard deviation
SET: short exercise test
