# Supplementary material for: The long exercise test as a functional marker of periodic paralysis
Source: Muscle Nerve. Author manuscript; Available in PMC 2023 Aug 14. (PMC7614949; doi:10.1002/mus.27465)
Supplement: Supplementary 1 [file EMS177440-supplement-Supplementary_1.docx]

**The long exercise test as a functional marker of periodic paralysis**

**Supplementary Material:**

**Statistical Analysis**

Shapiro-Wilk test and Levene’s test were used to assess normal distribution and homogeneity of variances, respectively. For comparisons between the 3 groups, One-way ANOVA was applied when assumption of normal distribution was confirmed (age and largest CMAP) and the non-parametric Kruskal-Wallis test was used when this assumption was rejected (percentage of CMAP increment). An unpaired Student’s *t*-test was used when comparing means of time to reach 40% decrement between the two groups with positive LET, assuming normal distribution and homogeneity of variance. Chi-squared test was used for comparison of categorical data (LET results vs. genotype; medication vs. LET result). Correlation of three ordered groups of binary data (frequency of attacks vs. LET results) was studied using the non-parametric Mann-Whitney U test. Gender differences amongst the groups were calculated using the Fisher’s exact test, due to small sample sizes (more than 20% of the expected values were less than 5).
